# Q Methodology in the COVID-19 Era

**DOI:** 10.3390/healthcare9111491

**Published:** 2021-11-02

**Authors:** Ahmed S. Alanazi, Heather Wharrad, Fiona Moffatt, Michael Taylor, Muhammad Ladan

**Affiliations:** 1 School of Health Sciences, University of Nottingham, Nottingham NG7 2HA, UK; heather.wharrad@nottingham.ac.uk (H.W.); Fiona.Moffatt@nottingham.ac.uk (F.M.); michael.g.taylor@nottingham.ac.uk (M.T.); 2 College of Medical Rehabilitation Sciences, Taibah University, Medina 42353, Saudi Arabia; 3 Faculty of Allied Health Sciences, Bayero University, P.M.B. 3011, Kano 700241, Nigeria; maladan.nur@buk.edu.ng

**Keywords:** Q methodology, methodology, online studies, COVID-19, data collection

## Abstract

All face-to-face studies were affected by the COVID-19 pandemic, as they could not be run in person due to rules and guidance linked to social distancing which were in force during the outbreak. Finding and testing an available COVID-secure approach for both participants and researchers was important as was the need to continue conducting such studies during this critical time. At present, the extant literature indicates a clear gap in research that elucidates how to carry out a Q methodology study online, step by step. This paper describes an option for online Q methodology using an approach that simulates all of the steps performed in a face-to-face setting using an open-source software known as Easy-HtmlQ. Using a case study in telemedicine adoption as illustration, this paper also considers the perspective of both research participants and Q methodology researchers via semi-structured interviews. Using Easy-HtmlQ V1.1 in online Q methodology studies appears to be an affordable, practical and user-friendly solution. Some of the benefits associated with running Q methodology studies online were the decreased costs, enabling the recruitment of wider number of participants, providing a COVID-19-secure environment and offering convenience to both participants and researchers during the research process. The findings of this study may contribute to increasing the number of online Q methodology studies in the future, as it has succeeded in offering a feasible approach for Q methodology researchers.

## 1. Introduction

The COVID-19 pandemic has acted as a catalyst for considering novel approaches to collecting research data due to the restrictions imposed on face-to-face activities. This has led to the use of digital tools for conducting interviews and focus groups [1]. In this paper, the conversion of the data collection phase of Q methodology to online is outlined using a case study in physiotherapy to describe the process. Using an online approach and digital technology in conducting a Q methodology study seems to present a practical and helpful solution where study participants can perform the study remotely without having to physically meet the researchers in person. Before describing the online Q methodology approach, the next two sections describe the origins of Q methodology and the steps that are involved in a face-to-face setting.

### 1.1. Overview of Q Methodology

William Stephenson, holding a PhD in both Physics and Psychology, created Q methodology in 1935 [2]. The letter ‘Q’ refers to the form of factor analysis that is applied in the methodology when analysing the data [3]. This methodology is designed to investigate the subjectivity in a systematic manner [4,5,6]. Q methodology has been used in many areas of research, such as healthcare [7,8,9,10], education [11,12], business [6], transportation [13], environment and agriculture [14].

The methodology differs from qualitative approaches that rely mostly on the participants’ narrative ability in expressing their viewpoints about a topic. With Q methodology, the participant is engaged in sorting/arranging pre-constructed statements according to his or her preference in a forced distribution [4] which is subsequently subjected to factor analysis so as to uncover varying viewpoint/subjectivities regarding a topic of discourse [3,15,16].

Q methodology usually recruits a small sample of participants which, it has been suggested, can adversely affect its ability to generalise findings [6,17]. However, Van Exel and De Graaf [6] argue that generalising the findings is not a concern of Q methodology, as the findings are represented viewpoints of a particular group of people which do not correlate directly with their number. In this regard, Thomas and Baas [18] used the term substantive inference to suggest that generalization in Q studies focus on concepts/categories, theoretical propositions and models of practice [3]. It is this that Brown [4] insisted that generalizations in Q are best thought of in terms of specimen and type. In light of this, Ref. [3] claim that Q methodology still has a broad usage and appeal, as it is concentrated on the concept of semantics in generalising the results, instead of statistics.

### 1.2. Process of Conducting a Q Methodology Study (Q Study)

**Figure 1:**
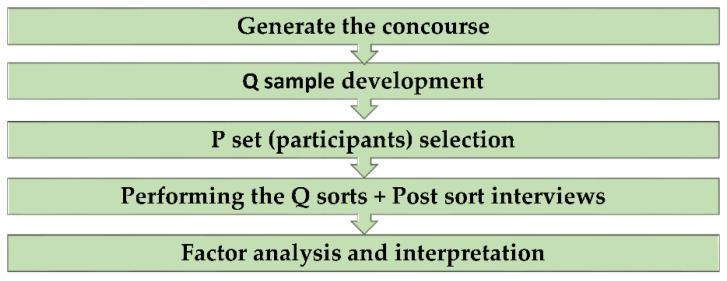
A summary of the process of conducting a Q study [23].

It is important to state that the process and steps of conducting a Q study are broadly the same whether the study is completed in a traditional face-to-face manner or online. The process of conducting a Q study (Figure 1) begins by formulating a research question which provides the focus for collating all related information about the topic; in Q methodology, this is referred to as the concourse. The concourse should be gathered from all available sources, such as published literature, public discussions within the topic, questionnaires, interviews and media [19]. Following this, statements are generated from the concourse, known as the Q set, which are delivered to the participants when doing the Q sort which refers to the activity, where participants rank the statement. When developing the Q sample, the researcher should try as accurately as possible to cover all aspects of the topic being explored, providing the participants with a clear and comprehensive representation without any gaps or overlaps [3].

The participants in the Q study are then selected; in Q methodology, they are termed the P set. It is important that there are sufficient numbers of participants in the P set to perform factor analysis, where viewpoints can be compared, contrasted and analysed [6]. As mentioned previously, participant numbers in Q studies tend to be quite small, as the statistical analysis does not rely on a very large number of participants [20]. Reference [4] suggests that the number of participants in the Q study should be between 40 and 60, whereas others encourage using a ratio between the number of statements (Q sample) and participants (P set). Some have suggested that there should be one participant to two statements [3]. Others have determined that the maximum ratio between participants and statements is one to three [16]. However, Brown (2010) in [21] argued against the use of such abstract rules, insisting that they originate from R-factor analysis. Therefore, as in other research methods, the researcher needs to decide on the optimum number of participants based on theoretical and pragmatic conditions for their study.

**Figure 2:**
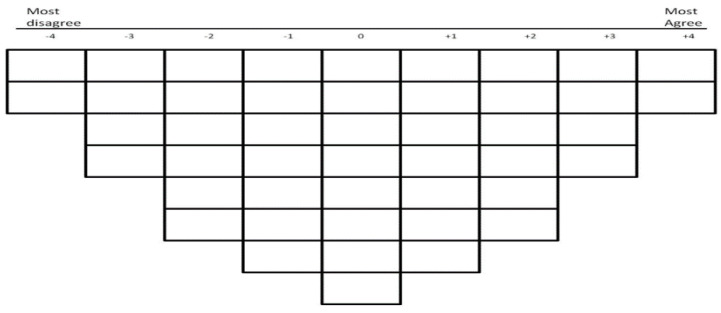
Grid for Q studies.

Once the Q sample and P set are established, the participants perform the Q sort. Participants arrange the Q sample, comprising a set of cards containing one statement per card, onto a custom grid, according to their viewpoints (Figure 2). The grid provides a bell-shaped arrangement of spaces ranging from what participants mostly agree with to those statements that they disagree with. The bell-shaped arrangement is designed to encourage the participants to arrange the statements in a sequential way according to their viewpoints. Furthermore, it also obliges them to make choices by having fewer spaces at the extremes (analogous to an Agree/Disagree Likert scale) but providing more spaces for more neutral responses [22]. After completing the Q sort, the participants’ grids are stored for future analysis. To complete the data collection phase of the Q study, an interview is conducted with participants in order to investigate the reasons behind their selection.

The final stage of the Q study is analysis and interpretation. The data from the Q sort activity are analysed using dedicated software programmes such as Ken-Q and PQMethod, which are generally recommended for analysing the data of a Q study [22]. The qualitative data from the post-sort interviews are considered as further data to inform and help the interpretation, as they are secondary data to the Q sort analysis which is the primary data set.

### 1.3. Moving to Online Q Methodology

Before COVID-19, the process of the Q study described above was typically completed in a face-to-face setting. However, face-to-face data collection during COVID-19 was not possible due to social distancing regulations. Therefore, it became imperative to find an alternative approach to Q methodology that was COVID-secure for both participants and researchers. However, there are currently no published studies that explain or describe the way of conducting Q methodology studies safely during COVID-19. Consequently, this paper presents an online Q methodology case study in telemedicine to illustrate and critique the online approach. This online case study simulates the same procedure that is followed in a traditional Q methodology study. It is also important to critically evaluate the usability of the technology used and the potential impact on the integrity of the data collected using this method. The paper also offers a pilot evaluation of the online Q methodology using interview data from two Q methodology experts and three participants who engaged in the online case study.

## 2. Materials and Methods

### 2.1. Study Aims

(i) To describe a solution for online data collection in a Q study and an evaluation of the usability and integrity of the approach.

(ii) To consider the benefits and drawbacks of remote data collection in a Q study.

### 2.2. Case Study: Developing the Online Q Study

The case study in this paper is part of a PhD project at the University of Nottingham. The case study aimed to evaluate the acceptability of telerehabilitation approaches by physiotherapists in Saudi Arabia. Implementing Q methodology online is a challenging task, as it requires simulating all steps that are performed in the face-to-face settings. During the COVID-19 pandemic, participation in Q methodology studies was achievable only through the use of online Q methodology software, which had to be affordable, manageable and intuitive for the users.

To this end, a number of software solutions were reviewed, but finally an open-source tool called Easy-HtmlQ V1.1 (freely available via the GitHub repository for open software development) was selected [24]. The software provides complete user documentation and simple editing of included Extensible Markup Language (XML) files allowing for easy customisation of the application for project purposes. Easy-HtmlQ is fully compliant with Google Firebase, which is a powerful and secure, free to use mobile application development platform for creating databases. Google Firebase provides secure database storage facilities once a user has completed the study and submitted their data. Google Firebase is password protected but easily accessible by the researcher using access credentials. The researcher can access all submitted data within Google Firebase and export the data out as a JSON (JavaScript Object Notation) file. JSON files are used to store simple and small quantities of data, and they can be modified with a text editor because they use open standard file formats; they can then be imported easily by other applications such as analysis software. In this case an open-source software called Ken-Q analysis was used to analyse the data imported from the JSON file [24]. The Ken-Q analysis provided the statistical output for the data set collected from participants’ Q sorts. The study information and consent form for the case study were sent via email to the study participants.

**Figure 3:**
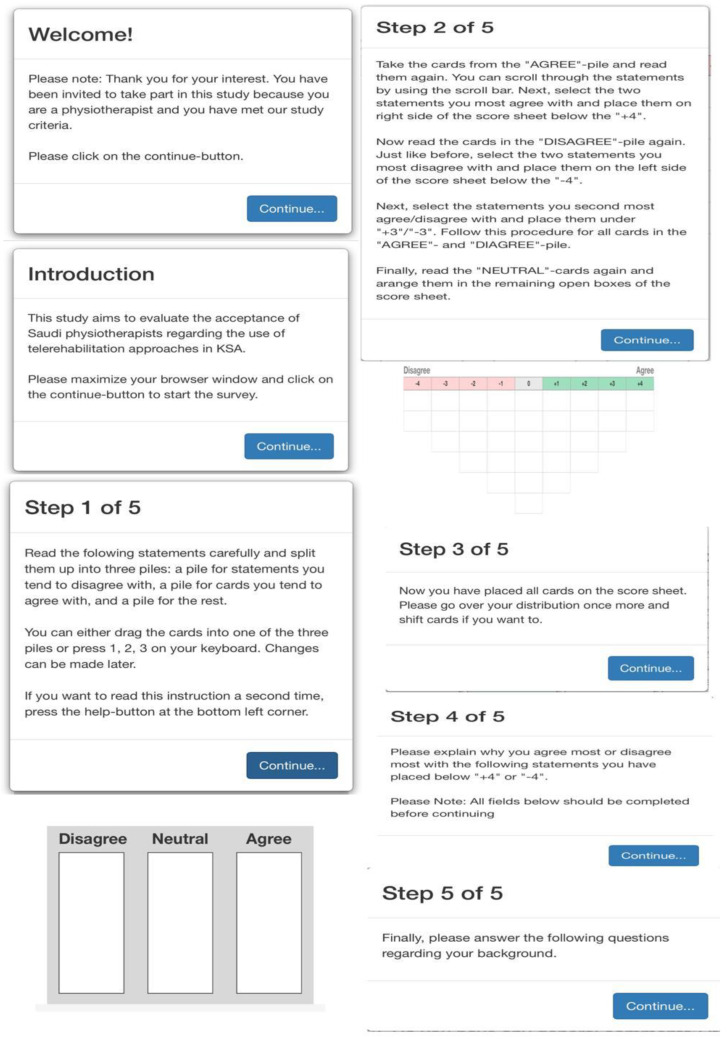
The steps presented to the participants.

The participants had to read all the study details and were asked by the end of the file to choose between (NEXT—I consent to take part or EXIT—I do not give consent). Participants who consented were then able to join the case study page. No personal logging details were required or downloading of any external Apps. A description of the project, together with guidelines on software use, were provided for participants on the opening pages. The guidelines were put in small windows that appeared to the participants before reaching each step in the case study. These windows included clear instructions for the participants in terms of what they needed to do in each step of the study. In each step, the participants had to complete the required action. The participants were able to go back to any previous step in the study and continue the study again from that point. Figure 3 shows the steps presented to the participants.

In traditional face-to-face Q studies, the post sort interviews are usually conducted after the Q sort activity. It is suggested that the interview should be done immediately after the Q sort, as the participant has to express the reasons behind their selections in the Q sort [3]. However, in the online Q methodology, it is difficult to ensure that every participant is interviewed soon after completing the Q sort activity online. The time factor is significant because, if the interview does not happen immediately after participants answer the Q sort, there is a strong possibility that they will forget their answers and rationale for their choices. Therefore, the online Q methodology used open-ended questions to be answered immediately following the Q sort as an alternative to the traditional interviews.

### 2.3. Pilot Evaluation of the Online Q Methodology

It is important to consider the perspective of both participants and Q methodology researchers regarding the process of conducting and participating in an online Q methodology study. Therefore, three participants (P1–P3), who had already participated in the online Q-methodology telemedicine case study, were volunteered to participate in semi-structured interviews in order to explore their experiences of taking part in a Q study online approach (Table 1). Furthermore, two experienced Q methodology researchers (P4 and P5) volunteered to complete the same online Q-sort as the case study participants to experience doing a Q-sort online. They then participated in the semi-structured interviews in order to evaluate the online platform and study process from the perspective of a researcher (Table 1).

The semi-structured interviews (*n* = 5) were conducted online and were audio-recorded. The analysis of qualitative data from the semi-structured interviews was carried out using [25] thematic analysis. The data gained from the interviews were organized in tabular format in order to make sense of them [26]. After all interviews were transcribed, the next stage involved searching for similar information from the participants’ responses to generate the main themes in a single document [25]. Expressions showing similar information were highlighted to aid identification. The developed themes were checked repetitively to conclude whether it was acceptable to split, merge or rename similar themes containing similar information [25,26,27].

## 3. Results

Four themes were identified: convenience, recruitment, cost and challenges.

### 3.1. Convenience

All participants in the semi-structured interviews believed that doing Q methodology online was more convenient than face-to-face Q methodology. For example, P1 thought that completing an online Q study provided more convenience in terms of time and space:

“The online experiment is more convenient, more handy, I can do it through my phone at my workplace or at my home at any time. So it will give me the opportunity to do it in my best time and my convenient place in any part of the day.”

This comment was supported by P2, who mentioned that completing the Q online could be accomplished while doing some other informal activities:

“The setting was very relaxed. I took my cup of tea, and I answered all the questions without any interference from any other circumstances.”

Both P4 and P5 believed that conducting Q methodology online could reduce the researcher’s workload; they implied that the online approach could help the researcher to collect and analyse data with less effort and time. P4 commented that:

“In face to face, once they finish doing the sorting, you might want to copy it into a shorter template, maybe an A4 to document what they have done. So that you will transfer that into the system when you want to do analysis. But with online Q methodology, as soon as they do, it is already there.”

P5 stated that:

“It will be easier and convenient for them to do your study. And also, for the researcher to do it online, it’s much easier and much organized and much more easy to pull the data.”

### 3.2. Recruitment

All participants thought that doing the Q methodology online would generally facilitate participant recruitment compared with face-to-face studies. For example, P1 highlighted the difficulty related to transport and time in face-to-face studies. P1 said that:

“The most important point for me, it’s the opportunity for me to be a part of this experiment because if it was face to face, I didn’t think so I will be able to be a part of it because it’s difficult for me to commute and meet you face to face.”

P2 thought the outbreak of COVID-19 provided the catalyst and opportunity for the Q methodology online rather than the traditional approach. P2 said that:

“If you asked me to participate face to face during the beginning of COVID-19, I will say I’m sorry, I can’t, because I was very conservative about the whole thing about COVID-19.”

P3 and P4 believed that the online studies generally helped to enable wider (global) participation in a time efficient way, and to allow the participation of those who could not attend face-to-face studies due to any reason. P3 mentioned that:

“You can involve as many people as you can from different countries, you are not restricted by the place.”

P4 stated that:

“I think online Q is seamless because in just one night, it can be sent across the world to get data and you can get the responses back within the shortest possible time.”

P5 reiterated the comments of P2, suggesting that conducting Q methodology online was the most appropriate way for completing data collection during the COVID-19 pandemic. P5 stated that:

“I think it is the only way and better way to do during these days due to the social distancing and all these measures to prevent COVID from spreading out.”

### 3.3. Cost

The Q methodology researchers’ opinions (participants 4 and 5) varied regarding whether conducting Q methodology online would decrease the financial cost of conducting Q methodology studies. Participant 5 thought that online Q methodology studies would make resource utilisation more efficient:

“It is going to reduce a lot of time waste and a lot of money waste.”

However, participant 4 provided a different opinion. P4 claimed that the money and resources, which are normally used for logistics, could be used to pay for participants’ internet data which allows them to participate in the study online:

“It is just that you have not used money for logistics in terms of transportation but that has now been pushed to the participant who is not going to use their own data. It depends on where you are going to do the study. But I think in developed settings, like where I did my study and where I am right now, you will still use that particular money.”

### 3.4. Challenges

All participants raised some concerns about conducting Q methodology studies online. For example, P1 highlighted the importance of providing clear instructions for completing Q methodology online. P1 perceived that providing such instructions could help to develop better understanding from the participants. P1 commented that:

“I think online questions should be done more carefully. It has to be clear for everyone. All participants have to understand it. Many people, I think are afraid to join the online version studies because they think it’s difficult or complicated or they cannot ask a person. But what I experienced with your experiment was the process was easy and clear.”

P2 and P3 agreed that internet could be a potential challenge in conducting the Q methodology online. The former stated that:

“If the internet connection is bad, it will affect the participants.”

The latter commented that:

“Nothing that happened to me, but I would consider if the internet connection was not very good. Maybe that would make me uncomfortable. But it’s something to consider. It is so worrisome to think about it.”

P4 believed that the most difficult challenge in conducting research in this way would be the technical aspect related to the development of Q methodology platform:

“I think, the difficult part is building the platform from the scratch, once you pass it, I think everything is easier because it is your work.”

P5 raised an essential concern over the research ethical considerations. P5 believed that it was important for both the researcher and the platform to protect each participant’s personal information. P5 stated that:

“It might be privacy issues especially with online studies, especially for us sensitive topics. The participant might seem not happy to discuss it online.”

## 4. Discussion

Conducting face-to-face research including Q methodology studies became very restricted during the COVID-19 pandemic. However, using online platforms to conduct Q methodology studies appeared to offer a practical solution that enabled these studies to continue during the pandemic. Whilst there are obvious disadvantages of conducting studies online rather than face-to-face, in this small study, several benefits were also gained when conducting Q methodology studies online rather than face-to-face. These could be summarised as lower costs, enabling wider recruitment and convenience for all involved.

There are some studies in the literature that have focused on online Q methodology [28,29,30,31,32]. However, the focus of these studies was on the topic of the study and not on the online Q methodology process itself. No studies have reported on the experience of Q methodology researchers or participants who used the online Q platform while conducting the study. Therefore, this study chose to focus on two aspects which firstly involved elaborating on how to develop Q methodology studies online through Easy-HtmlQ V1.1 software and secondly tried to garner the views/opinions of both researchers and participants who had used the Q-study software in order to highlight the negative and positive aspects from the viewpoint of all end-users.

Davis and Michelle [28] have claimed that the online Q methodology approach can reduce costs and increase reach/scope and recruitment, all of which are broadly analogous with the findings of this study. However, in terms of cost, this study noted that cost may be comparable with the face-to-face approach, particularly when factoring in having to pay for internet data for participants to be involved in the study. Van Exel and his colleagues [29] have used an online Q methodology version to collect their study data online, where they were able to receive participation from 10 different countries. The findings in this study support those of [28,29] regarding the ability of the online methodology to facilitate the recruitment of participants in Q methodology studies. Moreover, Van Exel and his group [29] and Jeffares and Dickinson [30] have used an online Q methodology version to collect their study data and were able to carry out post-sort interviews after doing the Q sort online. This way of conducting online Q methodology studies however does not seem to be practical because if the interviews do not happen immediately after performing the Q sort, it is likely the participants would have forgotten what they had done in the Q sort. Consequently, in an attempt to resolve this issue, this study used open-ended questions instead of interviews, where the participants successfully offered explanations about their selections of Q sort activity straight away, which in turn decreased the possibility of missing or forgetting any important information or points linked to the topic.

Walker and his group [31] tested a software called Q-Perspectives, and Lutfallah and Buchanan [32] tested a software called QMethod; both of these allowed participants to complete the Q sort on a computer instead of relying on paper-based data collection. Their aim was to reduce the Q methodology researcher workload when collecting and analysing data, as those researchers who use the face-to-face Q methodology have to initially record the participants’ answers in the Q sort activity and then manually enter all the data gathered into the analysis software. They also tried to reduce the possibility of data being recorded incorrectly by the researchers. Both studies found that using online software saved the researchers time as well as potentially reducing the likelihood of data errors being input into the analysis software. These findings also mirror those in this study in terms of the convenience that is realised as a result of using online software to collect Q methodology data. However, it should be noted that both studies tested this in a face-to-face setting where the participants and researchers were in the same location and the participants used smart devices to test the Q methodology software.

There are some generic challenges of online research that are not unique to Q methodology online. These issues concern the clarity of study guidelines, internet connection, technical issues linked to the platform, the ability to calculate the time for each participant and privacy of data [33,34,35]. Therefore, it is imperative for a research team to make the instructions of the study explicit and understandable, as it is not possible for them discuss these with the participants in real time [33,36]. Furthermore, the research team should possess the necessary skills/competence to assist with any technical issues that may be encountered during the study, such as checking internet connection quality with participants beforehand [33]. It is an important element to evaluate whether or not each participant was able to give the study a reasonable amount of time by using tools that enable a calculation of the time taken by each participant in the study [37]. Finally, it is important for researchers to assure the privacy of the data and reassure participants about data security, confidentiality and anonymity [38].

## 5. Conclusions

The COVID-19 pandemic was responsible for dramatic changes in the way we carry out research. Amidst this, Q methodology researchers were compelled to find a feasible way to conduct Q methodology studies, as conducting Q sorts face-to-face was no longer possible. Our study has shown that both participants and researchers found the online approach to be acceptable and, in some respects, preferable to the conventional face-to-face delivery. The Easy-HtmlQ v1.1 software integrated within a custom adaptation of the Google Firebase database had good usability for the participants and researchers. In addition to endorsing the technical fidelity and usability of the system used, other generic advantages of conducting studies online were reported. These were cost reductions, enhanced participant recruitment and greater convenience to both participants and researchers during the research process. This study provides an endorsement of the integrity of data collected using an online Q study, giving us confidence to carry out further studies using the same technical platform and instructions.

## Figures and Tables

**Table 1:** Participants’ overview.

Participant 1 (P1) Participant 2 (P2) Participant 3 (P3)	Physiotherapists who participated in the telemedicine case study
Participant 4 (P4) Participant 5 (P5)	Q methodology researchers

## Data Availability

Data are contained within the article.
